# A novel liver cancer diagnosis method based on patient similarity network and DenseGCN

**DOI:** 10.1038/s41598-022-10441-3

**Published:** 2022-04-26

**Authors:** Ge Zhang, Zhen Peng, Chaokun Yan, Jianlin Wang, Junwei Luo, Huimin Luo

**Affiliations:** 1 School of Computer and Information Engineering, Henan University, Kaifeng, China; 2 Present Address: Henan Key Laboratory of Big Data Analysis and Processing, Henan University, Kaifeng, China

**Keywords:** Cancer, Cancer screening, Computational biology and bioinformatics, Classification and taxonomy, Computational models, Machine learning, Microarrays

## Abstract

Liver cancer is the main malignancy in terms of mortality rate, accurate diagnosis can help the treatment outcome of liver cancer. Patient similarity network is an important information which helps in cancer diagnosis. However, recent works rarely take patient similarity into consideration. To address this issue, we constructed patient similarity network using three liver cancer omics data, and proposed a novel liver cancer diagnosis method consisted of similarity network fusion, denoising autoencoder and dense graph convolutional neural network to capitalize on patient similarity network and multi omics data. We compared our proposed method with other state-of-the-art methods and machine learning methods on TCGA-LIHC dataset to evaluate its performance. The results confirmed that our proposed method surpasses these comparison methods in terms of all the metrics. Especially, our proposed method has attained an accuracy up to 0.9857.

## Introduction

Liver cancer is the main malignancy worldwide, and its incidence is still increasing annually^1^. According to GLOBOCAN 2020, liver cancer causes about 830,000 deaths, ranking third leading cause of cancer deaths in 2020^2^. Studies have shown that early diagnosis of cancer can help improve survival rates^3^. However, the symptoms of liver cancer in early stage are not obvious^4^, most liver cancer patients are already in the middle and late stages when they are diagnosed, and treatment options are limited^5^. These factors causes that the liver cancer has a poor prognosis^6^. Therefore, it is of great practical importance to design a method that can effectively perform early diagnosis and help improve the treatment outcome of liver cancer.

With the emergence of gene sequencing technology, the amount of biological data has exploded^7,8^, which has provided researchers with plenty of omics data from different aspects, such as proteomics, transcriptomics, epigenomics, and genomics. Analyze and utilize these omics data for cancer diagnosis is a hot issue^9–12^. The cancer diagnosis methods can be normally categorized into two kinds, machine learning methods and deep learning methods. Sun et al.^13^ developed an improved feature selection method, called I-RELIEF, to extract hybrid features from breast cancer microarray data and clinical data. The extracted features were using to construct a breast cancer diagnostic model based on linear discriminant analysis (LDA). The excellent performance of the cancer diagnostic model was verified by comparing with several benchmark methods. Akay et al.^14^ proposed a breast cancer diagnosis method using SVM and F-score^15^. They first ranked the features by F-score, and then carried out grid search method to find parameters for SVM model which can get the best performance. Final experiment results indicated that this method had a better performance compared with previous works. Tsai et al.^16^ developed an artificial bee colony algorithm (ABC) combined with SVM for cancer diagnosis. They applied ABC to screen relatively important genes in gene expression data for cancer stage diagnosis, and identified some genes that could be used as biomarkers for further study. To address the problem that the success rate of liver cancer diagnosis is not satisfactory, Zhang et al.^17^ introduced a hybrid cancer diagnosis method, which is based on SVM, incremental feature selection (IFS) and max-relevance and min-redundancy (mRMR). Firstly, mRMR was used to screen the gene expression data, then IFS was used for further selection of the screened features, and finally the obtained genes were input to SVM for liver cancer diagnosis. However, Machine learning methods have difficulty processing raw data directly, they usually transformed the raw data into appropriate feature vectors. This may bring additional computational cost^18^.

Recent years, deep learning, which has the ability to capture intricate structures from raw data, started to gain attention in bioinformatics field and many cancer diagnosis methods based on deep learning method have been proposed^19^. Fakoor et al.^20^ reduced the dimensionality of gene expression data by principal component analysis (PCA). Then sparse autoencoder (SAE) was used for further feature extraction and finally softmax was used for cancer diagnosis. lyu and Haque^21^ transformed the gene expression data into 2-D images, then input the 2-D images into convolutional neural network to classify cancer of 33 tumor types. Gao et al.^22^ introduced a novel cancer diagnosis method (DeepCC). DeepCC performs gene enrichment analysis to transform the gene expression data into functional spectra. Then the resulting functional spectra are input into a multilayer neural network for subsequent training. For both colorectal and breast cancer, DeepCC outperforms random forest (RF) and SVM for cancer subtype classification. However, previous deep learning-based models mainly use single omics data, which is limited to describe all the features of cancer^23^. It limits the performance of deep learning in cancer diagnosis.

Accordingly, cancer diagnosis methods based on multiple omics data are increasingly adopted^24–26^. Sun et al.^27^ proposed a deep learning method which is based on model fusion, named MDNNMD, for breast cancer prognosis. They used two types of omics data, gene expression data and copy number variation (CNV), as well as clinical data, and constructed three deep neural network (DNN) models for the three types of data, and finally fused the prediction scores of the three independent models as the final prediction result. Zhang et al.^28^ used variational autoencoder (VAE) to integrate methylation data and gene expression data to diagnose cancer. They used ten-fold cross-validation on 33 types of cancers to evaluate their method, and the final accuracy obtained by their method is 97.49%. Copy number variation, gene expression, and methylation data were used in these researches on cancer diagnosis. This indicated that copy number variation, gene expression, and methylation data bring useful information to cancer diagnosis. Thus, all these three omics data were selected in this work.

Previous studies have often only used genomics data. Interpretability is particularly required in genomics because of relatively smaller sample sizes and to better understand the molecular causes of disease so that targeted therapies can be designed^29^. Patient similarity network (PSN) can address these problems and specializes in integrating multi-omics data and generating interpretable models^30^. However, previous works rarely took the patient similarity into account. To address this issue, we integrated three omics data of liver cancer and calculated the similarity between patients. As the similarity network is none-Euclidean data, previous neural networks like CNNs, are hard to handle this data^31,32^. Thus, graph convolutional network (GCN), which has the advantages in processing non-Euclidean data is used in this work. Meanwhile, since omics data have small sample size, we need a deeper network to fit the data and thus avoid the disadvantages associated with the small sample size^33^. But the number of GCN layers is rarely more than four because of the vanishing gradient problem^34^. To deal with this challenge, we selected the dense graph convolutional neural network (DenseGCN)^35^. DenseGCN improves information flow in the network by densely connecting different layers. DenseGCN is able to overcome the vanishing gradient problem and make the GCN architecture deeper, thus enabling better utilization of patient similarity network and multi-omics data for cancer diagnosis. To the best of our knowledge, this is the first effort to employ DenseGCN in cancer diagnosis field.

In this work, A novel liver cancer diagnosis method (pDenseGCN) based on patient similarity network and DenseGCN is proposed. We first used similarity network fusion (SNF) to construct the patient similarity network using three liver cancer omics data. Then, we extracted latent embedding representation of omics data by using denoising autoencoder (DAE). This can provide a more precise representation of liver cancer. Finally, we adopted DenseGCN for liver cancer diagnosis based on the patient similarity network and latent representation of omics data. By incorporating the supplemental information PSN into the model, we got a more comprehensive view of cancer and finally obtained better performance on liver cancer diagnosis. According to the reliable experiments, our method pDenseGCN gained an accuracy score of 0.9857, and performed better compared with five state-of-the-art methods and machine learning methods.

The main contributions of this paper are as follows.

- A novel deep learning method, named pDenseGCN, is proposed for effectively liver cancer diagnosis.
- pDenseGCN utilizes SNF to construct a patient similarity network based on multi-omics, thus captures the similarity information between patients, which helps in liver cancer diagnosis.
- pDenseGCN adopts DenseGCN as the classifier. DenseGCN connects different layers densely to improve information flow in the network, which can overcome vanishing gradient problem. This brings better results in liver cancer diagnosis.

## Methods

### Proposed method

**Figure 1:**
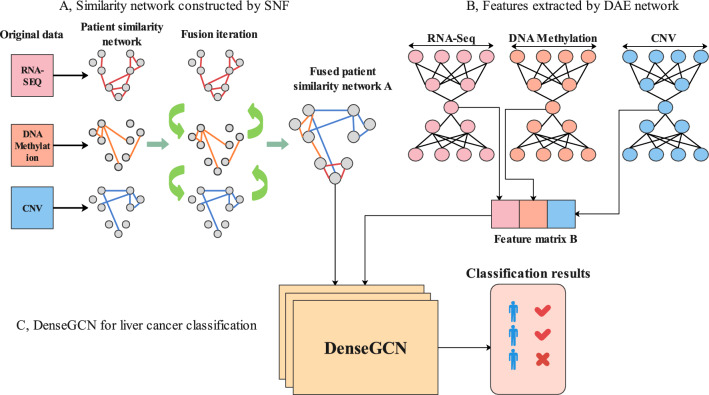
The overall workflow of pDenseGCN. (**A**) Similarity network constructed by SNF. (**B**) Features extracted by DAE network. (**C**) DenseGCN for cancer diagnosis.

There are three components in the proposed method pDenseGCN. The first component is generating patient similarity network by omics datasets. Three omics datasets were applied as the input of similarity network fusion method to produce patient similarity network. The second component is extracting feature by denoising autoencoder. In this step, RNA-Seq, DNA Methylation and CNV were put into denoising autoencoder respectively to obtain low-dimensional features. The next component is to input the obtained patient similarity network and feature matrix into dense graph convolutional network (DenseGCN) for classified training and prediction, and a cancer prediction framework was finally built. Figure 1 describes the overall workflow of our proposed method pDenseGCN.

### Construction of patient similarity network

In order to construct the patient similarity network (PSN), we employed a method named Similarity network fusion (SNF), which can make full use of multi-omics^36^. SNF is applied to combine RNA-seq, DNA methylation and CNV data to generated a patient similarity network. Assuming that there are n patients, each of them has m type data (such as RNA-Seq and DNA methylation). We represent the PSN as a graph G=(V,E), where V represents the set of patients {x$_{1}$, x$_{2}$, x$_{3}$..., x$_{n}$} and the edges E correspond to the similarity between vertices v $\in$ V. The weights between edges are represented by an n $\times$ n similarity matrix W which is computed by Eq. (1).

1 $$\begin{aligned} {{W}_{i,j}}=\exp (-\frac{{{\phi }^{2}}({{x}_{i}},{{x}_{j}})}{\alpha {{\gamma }_{i,j}}}) \end{aligned}$$

where $\alpha$ is a hyperparameter, $\phi$ (x$_{i}$, x$_{j}$) is the Euclidean distance between patients x$_{i}$ and x$_{j}$ and $\gamma$ $_{i,j}$ is used to eliminate the scaling problem. In order to compute the fused matrix from multiple types of data, the similarity matrix is normalized as Eq. (2).

2 $$\begin{aligned} {{P}_{i,j}}= {\left\{ \begin{array}{ll} \dfrac{{W}_{i,j}}{2\sum _{k\ne i}{ {{W}_{i,k}}}} &{} j\ne i \\ \dfrac{1}{2} &{} j = i \end{array}\right. } \end{aligned}$$

Assuming N$_{i}$ is a set of x$_{i}$’s neighbors. Then local affinity matrix S is calculated by Eq. (3).

3 $$\begin{aligned} {{S}_{i,j}}={\left\{ \begin{array}{ll} \dfrac{ {{W}_{i,j}}}{\sum _{k\in {{N}_{i}}}{ {{W}_{j,k}}}} &{} j\in {{N}_{i}} \\ 0 &{} otherwise \end{array}\right. } \end{aligned}$$

Let P$_{t}$ $^{(h)}$ represent normalized similarity matrix of h-th type data (1 $\le$ h $\le$ m) in the t-th iteration, P$_{t}$ $^{(h)}$ is updated according to Eq. (4).

4 $$\begin{aligned} P_{t+1}^{(h)}={{S}^{(h)}}(\dfrac{\sum \limits _{k\ne h}{P_{t}^{(k)}}}{m-1}) {{({{S}^{(h)}})}^{T}} \end{aligned}$$

where the S$^{(h)}$ represents local affinity matrix of h-th type data. Through this process of continuous iterative fusion, a patient similarity network which contains complementary information from three omics dataset is finally obtained. The fused network can be used for classification or clustering, and in this work the fused similarity network is taken as the input of DenseGCN for cancer diagnosis.

### Feature extraction by denoising autoencoder

To reduce the noise in the row omics data and the computational cost, we constructed three independent denoising autoencoders to extract latent embedding representation from the omics datasets, respectively. The autoencoder (AE) is a neural network which typically contains two networks: an encoder network and a decoder network. The encoder network takes a feature vector x $\in$ $\Re$ $^d$ as input and encodes it into a low-dimensional representation y $\in$ $\Re$ $^q$, define as *f*$_e$: x $\rightarrow$ y. The decoder network maps the low-dimensional representation y back to the input space, define as *f*$_d$: y $\rightarrow$ z. The autoencoder is optimised by minimizing the reconstruction loss L between original input x and reconstructed input z as Eq. (5).

5 $$\begin{aligned} \arg {{\min }_{{{f}_{e}},{{f}_{d}}}}L(x,z) \end{aligned}$$

where *f*$_e$, *f*$_d$ represent the parameters of the encoder network and the decoder network, respectively.

In this work a denoising autoencoder (DAE)^37^ is applied to extract latent embedding representation. The architecture of DAE is the same as AE, but the way to train network is different. DAE first corrupted the input data by adding noise, then the corrupted input data x$\_$noise is fed to the autoencoder. By recovering the damaged input data, DAE extracts robust latent embedding representation. We use the loss function Mean Squared Error to train DAE. The latent embedding representations extracted by three independent DAE are connected and then fed to the further work together with patient similarity network.

### DenseGCN

The patient similarity network constructed by SNF is non-Euclidean data that CNNs fail to handle^32^, so GCN is considered in this work because of their advantages in processing non-Euclidean data^38^. However, original GCN model is usually very shallow due to the vanishing gradient problem, this limits the ability of GCN to fit the data^35^. So an improved GCN model named DenseGCN is used in this work.

GCN takes a feature matrix X which describes every node in the graph and an adjacency matrix A which illustrate the structure of the graph as input and generates a node-level matrix Z. The layer-wise propagation rule of GCN can be formulated as Eq. (6).

6 $$\begin{aligned}H(N)=f(H(N-1),A)=\sigma (AH(N-1)W(N))\end{aligned}$$

where H(N) is the output of the N layer, and W(N-1) is a weight matrix of the N-1 layer. *f*($\cdot$) represents graph convolution operation. $\sigma$($\cdot$) is an activation function which is usually non-linear. This rule is valid but still has some limitations. Frist the feature vectors of all neighboring nodes are taken into consideration, but the node itself is ignored. This limitation can be fixed by adding self-connections to the adjacency matrix A, define as ${\hat{A}}$ = A+E, where E represents the identity matrix. The second limitation is that A is usually not normalized, this means that the scale of the feature vectors will change when multiplying with A. To get rid of this limitation, symmetric normalization, defining as *D*$^{-1/2}$AD$^{-1/2}$, is applied to standardize A, where D is the diagonal node degree matrix. Thus, propagation rule is reformulated as Eq. (7).

7 $$\begin{aligned}H(N)=f(H(N-1))=\sigma ({{\widetilde{D}}^{-\frac{1}{2}}}\widetilde{A}{{\widetilde{D}}^{-\frac{1}{2}}}H(N-1)W(N))\end{aligned}$$

**Figure 2:**
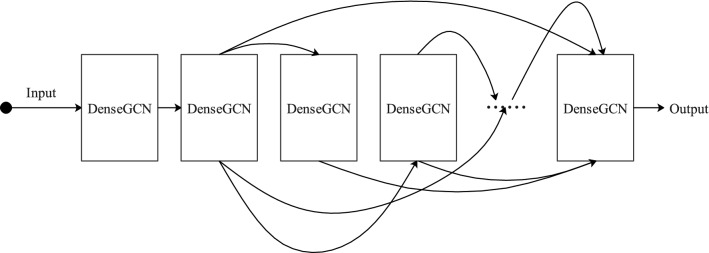
The structure of DenseGCN.

Theoretically, deeper networks are able to learn more abstract representations and require less data for training than shallow neural networks^33,39^, and at the same time, omics data are characterized by high dimensionality and few samples. This indicates that deep networks are more applicable to omics data. However, GCN is usually very shallow because of the vanishing gradient problem^35^, and most state-of-the-art GCNs are less than 4 layers^34^. Inspired by the dense connectivity of DenseNet^40^, a similar idea is adapted to GCN to improve information flow in the network and avoid gradient vanishing problem^35^. This dense model, named DenseGCN, has a new propagation rule which is define as Eq. (8).

8 $$\begin{aligned} H(N)=T(f(H(N-1),A),f(H(N-2),A),\ldots ,f(H(0),A)) \end{aligned}$$

where H(0) is the input feature matrix X, *T*($\cdot$) represents a vertex-wise concatenation function. The structure of DenseGCN is shown in Fig. 2.

In summary, the original GCN is limited by the gradient disappearance problem, which makes it difficult to have a deep network architecture. In contrast, DenseGCN improves the flow of information by connecting layers densely to solve the gradient vanishing problem, and is able to have a deeper network architecture compared with original GCN. Thus, DenseGCN is more suitable for omics datasets.

## Results

A series of experiments were conducted to evaluate the performance of proposed method pDenseGCN. First, pDenseGCN was compared with five state-of-the art methods, namely ASVM^41^, Xgboost-AD^42^, MGRFE-GaRFE^43^, ET-SVM^44^, XOmiVAE^45^, and four machine learning methods, namely Linear Discriminant Analysis (LDA), Naïve bayes (NB), Random Forest (RF), and Decision Tree (DT). Then we investigated the influence of patient similarity network and different omics data. Finally, we discussed the impact of different number of DenseGCN layers and features selected by DAE.

### Datasets and data preprocessing

We performed our proposed method pDenseGCN on Liver Hepatocellular Carcinoma (LIHC) omics datasets acquired from TCGA portal (https://www.cancer.gov/tcga). A R package named TCGA-assembler^46^ was used to obtain DNA methylation, RNA-seq and CNV data of LIHC. The detail of above three datasets is described in Table 1.

**Table 1:** The details of three omics datasets.

Omics type	Number of samples	Number of features
RNA-Seq	424	20,530
DNA methylation	429	20,421
CNV	760	24,924

Similar to the previous literature^47^, these three datasets are preprocessed by following steps. The first step is outlier removal. We delete these features which have more than 20% missing values. Similarly, these sample which have moved than 20% features have been removed. 404 common samples remained in this step. The next step is missing-data imputation. We use the mean of remaining features to impute the missing values based on the python package sklearn^48^. Finally, these three datasets are normalized according to Eq. (1).

9 $$\begin{aligned} {{X}_{nor}}&= \frac{X-{{X}_{min }}}{{{X}_{max }}-{{X}_{min }}} \end{aligned}$$

where X is any column in the omics dataset, X$_{nor}$ is the corresponding columns after normalization, X$_{max}$ is the maximum values in *X* and X$_{min}$ represent the minimum values in X.

### Evaluation metrics

To fully evaluate different methods, accuracy, precision, recall, F1-score^49^, and AUC^50^ were used as the metrics. All of them are defined as follows.

Accuracy: The ratio of correctly predictions. Accuracy can be calculated as Eq. (10).

10 $$\begin{aligned} Accuracy&=\frac{TP+TN}{TP+TN+FP+FN} \end{aligned}$$

Precision: The ratio of samples categorized as positive to those which are actually positive. The formula of precision is Eq. (11).

11 $$\begin{aligned} Precision=\frac{TP}{TP+FP} \end{aligned}$$

Recall: The ratio of true positive samples divided into positive samples. It is defined as Eq. (12).

12 $$\begin{aligned} Recall=\frac{TP}{TP+FN} \end{aligned}$$

F1-score: The harmonic means of recall and precision. It can be calculated as Eq. (13).

13 $$\begin{aligned} F1-Score=\frac{2 \times Recall \times Precision}{Recall + Precision} \end{aligned}$$

AUC: The area under the receiver operating characteristics curve.

### Experiment and parameter settings

For these omics dataset, 60% of the data was randomly selected to train models and 20% of the data was randomly selected as the validation set. The remaining 20% data was used for testing. To reduce the deviation, we repeated the experiments five times and the average result of the five experiments was taken as the ultimate result of the experiment. All of our models were implemented using Pytorch. The experiments were executed on a PC with an Intel core i7-10700 processor of 2.90 GHz and 32.0 GB RAM. The relevant parameters of the used methods are listed in this part. For pDenseGCN, we determined the optimal learning rate (Lr) and the batch size according to the grid search method. For the comparison algorithm, the parameters given in its original paper were slightly modified to make it more suitable for our dataset. Table 2 describes the detailed parameters.

**Table 2:** Parameter settings.

Methods	Parameters
pDenseGCN	Lr(DAE) = 0.01, epochs(DAE)=50, batch size(DAE)=8, Lr(DenseGCN)=0.01, epoch(DenseGCN)=500
ASVM	m=4, n=8, q=5, numGlobal=30, numLocal=20
Xgboost-AE	Lr(AE)=1.0, batch size(AE)=16, epoch(AE)=100
MGRFE-GaRFE	global_bestsize = 120, layer_bestsize = 100 , total_layer = 2
ET-SVM	C=0.004, kernel=‘linear’, decision_function_shape=‘ovo’, gama=1
XOmiVAE	learning_rate=0.01, dropout=0.5, epoch=100
LDA	solver=’svd’
NB	var_smoothing=1e-09
RF	n_estimators=10
DT	splitter=’best’, min_samples_split=2, min_samples_leaf=1

### Comparison with other methods

To validate the performance of our proposed method pDenseGCN, we compared it with five state-of-the-art methods and four machine learning methods. We replicated them according to their publications or using publicly available programs. The details of these five state-of-the-art methods are described below.

- ASVM^41^ is a novel multilayer recursive feature elimination algorithm based on embedded variable length encoding genetic algorithm aiming at cancer classification. It utilizes the Shuffled Frog Leaping algorithm to adaptively adjust the parameters of the Support Vector Machine based on data attributes to classify early stage cancers.
- Xgboost-AD^42^ is a novel cancer classification method. It integrates multi-omics data by autoencoder and utilizes extreme gradient boosting to accurately diagnostic classify cancer.
- MGRFE-GaRFE^43^ is aiming to use fewer genes for better cancer classification results. It applies a multilayer recursive feature elimination method based on an embedded genetic algorithm to get a better feature subset for cancer classification.
- ET-SVM^44^ adopts extra trees and variance threshold to select features from gene expression data, and uses these important features to diagnostic classify cancer based on SVM.
- XOmiVAE^45^ is an interpretable deep learning model for cancer diagnosis based on variational autoencoder. It uses variational autoencoder to extract low-dimensional expressions from genomics data, which are then fed into a multilayer perceptron for cancer classification.

The results are displayed in Table 3. As seen in Table 3, pDenseGCN has a better performance compared with other methods among all the metrics in LIHC dataset. In terms of accuracy, pDenseGCN achieves 98.57% accuracy, which is 1.31% better than the best remaining method XGBoost-AD and up to 23.9% better than other comparison methods. As for the other four metrics, pDenseGCN gains a best performance which are up to 26.03%, 35.49%, 22.46%, 24.09% better than other methods in terms of precision, recall, f1-score, and AUC. It proves that by introducing the patient similarity network, our proposed method is more advantageous in cancer diagnosis and more applicable to the LIHC dataset.

**Table 3:** Results of comparison methods and proposed method.

	Precision	Recall	F1-Score	Accuracy	AUC
pDenseGCN	**0.9865**	**0.9865**	**0.9865**	**0.9857**	**0.9856**
ASVM	0.937	0.9744	0.9553	0.9208	0.8531
XGBoost-AD	0.9736	0.9729	0.9732	0.9726	0.9759
MGRFE-GaRFE	0.9689	0.9397	0.9183	0.954	0.8306
ET-SVM	0.96	0.6316	0.7619	0.7945	0.8015
XOmiVAE	0.946	0.8974	0.9211	0.8537	0.8718
LDA	0.7262	0.8133	0.7673	0.7466	0.7447
RF	0.9605	0.9125	0.9359	0.937	0.9848
NB	0.8977	0.7914	0.8412	0.8452	0.8492
DT	0.9254	0.8267	0.8732	0.8767	0.8781

Significant values are in bold.

### The influence of patient similarity network

**Figure 3:**
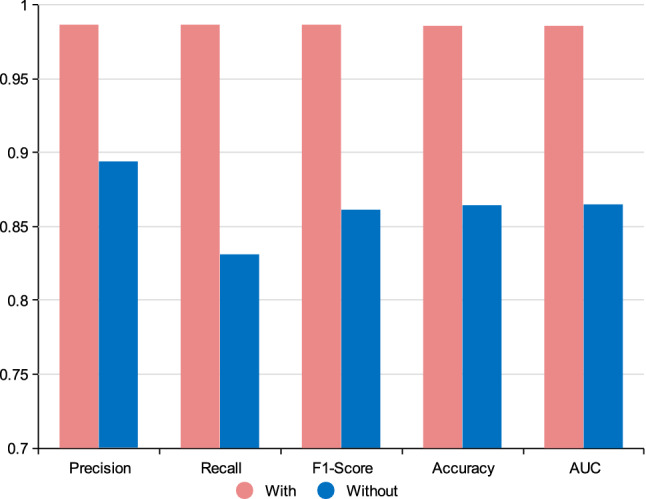
The influence of the patient similarity network.

Constructing patient similarity network is one important component of pDenseGCN, since the patient similarity network allows DenseGCN to gain information from the neighboring patients. To investigate the influence of patient similarity network on cancer diagnosis, we designed two experiments. One experiment took patient similarity network as the input and the other one took an identity matrix as the input. The results are presented in Fig. 3. As Fig. 1 shows, the model trained with patient similarity network performs prior to the model trained without patient similarity network. In the case of precision, recall, F1-score, accuracy, and AUC, the model trained with patient similarity network is 9.29%, 15.6%, 12.6%, 12.2%, 12.1% higher than the model trained without patient similarity network. This demonstrates that by introducing a patient similarity network, our proposed method pDenseGCN takes the information from neighboring patients into consideration when predicting the label of a patient. This effectively improves the classification results.

### Effectiveness of different omics data

We carried out experiments with varied type of data to confirm the effectiveness of varied omics data and the effect of multi-omics data combination. The results are displayed in Table 4. In Table 4, RNA-seq, DNA Methylation and CNV represent three single omics data, respectively. RNA-Seq+DNAMethy, RNASeq+CNV, and DNAMethy+CNV represent three omics data pairwise combinations, respectively. Multi-Omics represents our proposed method with three omics data. We can see from Table 4 that the performance of our proposed method rises over time as the type of data used increases. These models trained with single omics data have an accuracy of up to 0.8286, however, when the model was trained with two omics data, the lowest accuracy was 0.9. The optimal performance is attained when the model is trained with three kinds of omics data with an accuracy value of 0.9857. It confirms that multiple omics do outperform single omics, and that the performance improves progressively as the number of omics data increases. This indicate that different omics data contain complementary information, which provides a comprehensive view of cancer and improves the result of cancer diagnosis. Besides, the model trained with DNA Methylation performs better in the three single omics data, this may indicate that DNA Methylation contains more information that facilitates cancer diagnosis.

**Table 4:** Results of different omics data.

	Precision	Recall	F1-score	Accuracy	AUC
RNA-Seq	0.8197	0.6757	0.7407	0.75	0.7545
DNA methylation	0.931	0.7297	0.8182	0.8286	0.8346
CNV	0.9574	0.6081	0.7438	0.7785	0.7889
RNA-Seq+DNAMethy	0.9855	0.9189	0.951	0.95	0.9519
RNASeq+CNV	0.8375	0.9853	0.9054	0.9	0.9024
DNAMethy+CNV	0.9589	0.9459	0.9524	0.95	0.9502
Multi-omics	**0.9865**	**0.9865**	**0.9865**	**0.9857**	**0.9856**

Significant values are in bold.

### The effect of DenseGCN layer numbers

In order to explore the effect of different DenseGCN layer numbers on the final result, we designed several models with various number of layers. The results of different models are shown in Table 5. As seen in Table 5, unlike the conventional GCN models, pDenseGCN still performs well even if the number of layers is more than three. Meanwhile, excluding 7-layers and 8-layers, the performance of pDenseGCN increases gradually with the increase of layers. This illustrates that deep network is more suitable to fit omics data and can gain a better performance than shallow network in cancer diagnosis. When the number of pDenseGCN layers reaches 10, multiple metrics such as Recall, F1-Score, Accuracy and AUC perform best. However, as the number of layers keeps increasing, these scores of metrics gradually decline. This is probably because although DenseGCN overcomes gradient vanishing by densely connecting layers to some extent, the number of DenseGCN layers is not the more the better. Therefore, the number of layers of pDenseGCN in this work is set to 10.

**Table 5:** Results of different DenseGCN layer numbers.

	Precision	Recall	F1-Score	Accuracy	AUC
3-layers	1	0.6892	0.816	0.8357	0.8446
4-layers	0.8024	0.8784	0.8387	0.8214	0.8179
5-layers	1	0.9324	0.965	0.9643	0.9662
6-layers	0.9125	0.9865	0.9481	0.9429	0.9402
7-layers	1	0.7297	0.8437	0.8571	0.8649
8-layers	0.9667	0.7838	0.8657	0.8714	0.8767
9-layers	1	0.9459	0.9722	0.9714	0.973
10-layers	0.9865	**0.9865**	**0.9865**	**0.9857**	**0.9856**
15-layers	0.925	1	0.961	0.9571	0.9545

Significant values are in bold.

### The effect of feature numbers extracted by DAE

**Figure 4:**
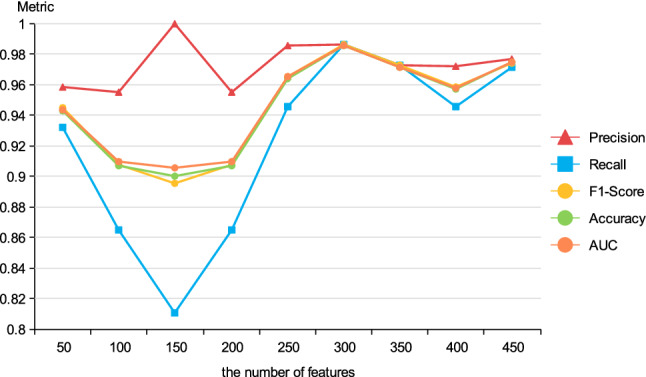
Results of different number of features.

To investigate the effect of feature numbers on model performance, we conducted several experiments with different number of features extracted by DAE to explore the changes in experimental results. The results are displayed in Fig. 4.

As can be seen from Fig. 4, when the quantity of features ranges from 100 to 300, three metrics F1-Score, Accuracy, AUC exhibit an obvious rising trend. This may be because that useful information that the model can learn gradually increases as the amount of features increases. The proposed method reaches best performance when the number of features is set to 300, with an accuracy value of 0.9857. As the number of features continues to grow, the performance of our proposed method begins to gradually decrease instead. This indicates that when the number of features is large, irrelevant or redundant information may be incorporated, which does harm to the performance of model. Thus, 300 is selected as the number of features in this work.

## Conclusion

Liver cancer is one of the common malignant tumors worldwide with a poor prognosis. Since effective diagnosis helps to improve the cure of liver cancer, there is an urgent need for a method that can accurately perform diagnosis of liver cancer. In this work, we establish a novel method pDenseGCN which consists of similarity network fusion, denoising autoencoder, and dense graph convolutional network for liver cancer diagnosis. The pDenseGCN takes multi-omics data to construct a patient similarity network, which brings more patient information for cancer diagnosis. We explore the differences in the results of pDenseGCN trained with and without patient similarity network. The results indicate that the similarity information does contribute to cancer diagnosis. In addition, since the patient similarity network is non-Euclidean data, and the omics data is characterized by high dimensionality and few samples, pDenseGCN utilizes densely connected graph convolutional neural network to fit them better. Compared with state-of-the-art methods, pDenseGCN achieves better results in terms of the final prediction performance metrics. It demonstrates that our proposed pDenseGCN is a promising method for liver cancer diagnosis. In our future work, we are committed to extend our proposed method to multi-classification tasks, such as cancer subtype classification as well as pan-cancer classification.
